# Neonatal Seizures: An Overview of Genetic Causes and Treatment Options

**DOI:** 10.3390/brainsci11101295

**Published:** 2021-09-29

**Authors:** Giulia Spoto, Maria Concetta Saia, Greta Amore, Eloisa Gitto, Giuseppe Loddo, Greta Mainieri, Antonio Gennaro Nicotera, Gabriella Di Rosa

**Affiliations:** 1 Unit of Child Neurology and Psychiatry, Department of Human Pathology of the Adult and Developmental Age “Gaetano Barresi”, University of Messina, 98125 Messina, Italy; giulia.spoto27@gmail.com (G.S.); mariasaia922@gmail.com (M.C.S.); agreta18@gmail.com (G.A.); gdirosa@unime.it (G.D.R.); 2 Unit of Neonatal Intensive Care, Department of Human Pathology of the Adult and Developmental Age “Gaetano Barresi”, University of Messina, 98125 Messina, Italy; egitto@unime.it; 3 Azienda USL di Bologna, 40124 Bologna, Italy; loddogiuseppe0@gmail.com; 4 Department of Biomedical and NeuroMotor Sciences, University of Bologna, 40138 Bologna, Italy; greta.mainieri2@unibo.it

**Keywords:** benign familial neonatal epilepsy, early myoclonic encephalopathy, early-infantile epileptic encephalopathy, epilepsy, genotype-phenotype correlation, neonatal epilepsy, neonatal seizures

## Abstract

Seizures are the most frequent neurological clinical symptoms of the central nervous system (CNS) during the neonatal period. Neonatal seizures may be ascribed to an acute event or symptomatic conditions determined by genetic, metabolic or structural causes, outlining the so-called ‘Neonatal Epilepsies’. To date, three main groups of neonatal epilepsies are recognised during the neonatal period: benign familial neonatal epilepsy (BFNE), early myoclonic encephalopathy (EME) and ‘Ohtahara syndrome’ (OS). Recent advances showed the role of several genes in the pathogenesis of these conditions, such as *KCNQ2*, *KCNQ3*, *ARX*, *STXBP1*, *SLC25A22*, *CDKL5*, *KCNT1*, *SCN2A* and *SCN8A*. Herein, we reviewed the current knowledge regarding the pathogenic variants most frequently associated with neonatal seizures, which should be considered when approaching newborns affected by these disorders. In addition, we considered the new possible therapeutic strategies reported in these conditions.

## 1. Introduction

Seizures represent the most frequent neurological event during the neonatal period, reflecting a central nervous system (CNS) dysfunction. They occur in 1 to 4 *per* 1000 live births during the first days of life, with a significantly higher rate in preterm babies [1,2,3,4]. The immature brain, with its peculiar neuronal network properties and continuous developmental modifications, appears to be particularly hyperexcitable, hence, more prone to produce seizures than in any other period of life [1,5].

In neonates, video-electroencephalogram (EEG) recording is the gold standard for the diagnosis [6]. As seizures in the neonatal period have been shown to have a focal onset, their classification into focal and generalised is unnecessary [7]. However, seizures can associate to motor (i.e., automatisms, clonic, epileptic spasms, myoclonic) or non-motor (autonomic or behaviour arrest) clinical manifestations [7].

Neonatal seizures etiopathogenesis can be ascribed to an acute event (such as hypoxic-ischemic encephalopathy, intracranial haemorrhage, stroke, infection or electrolyte disturbances) or symptomatic conditions (namely genetic, metabolic or structural ones), outlining the so-called ‘Neonatal Epilepsies’ [1,8].

Nevertheless, it is frequently arduous to make a rigid etiological distinction between genetic, metabolic and structural forms. Metabolic disorders, as well as malformations (structural conditions), have in fact a genetic cause, even though detecting the causative pathogenic variant is not always easy. Uncoupling the aetiologies may be therefore difficult and even improper [8]. In this regard, the latest International League Against Epilepsy (ILAE) Task Force on Neonatal Seizures [9] proposed a reorganised, less rigid and non-hierarchical diagnostic classification. It is possible to taxonomize epilepsies in more than one category (genetic, metabolic, structural, immune and unknown) which are no longer considered mutually exclusive. Since genetic, metabolic and structural causes explain the 10–15% of all neonatal seizures, this approach is useful in providing an appropriate diagnostic assessment, allowing an adequate and early treatment initiation and preventing poor outcomes [1,2,4,7,8,10].

According to a more traditional, syndromic categorisation, the ILAE identified the most common forms of neonatal epilepsies, namely the ‘Benign familial neonatal epilepsy’ (BFNE), the ‘Early myoclonic encephalopathy’ (EME) and the ‘Ohtahara syndrome’ (OS) [2,9]. Nowadays, these conditions are mostly considered genetic disorders, even when a metabolic or structural cause is revealed, strongly supporting the importance of the application of the ILAE classification. Monogenic mutations could result in a loss of function (Lof) or a gain of function (Gof) of the gene product, which may be detected in most of these cases [2,8]. Different pathogenic variants within the same gene may result in opposite effects (LoF or GoF) on the gene product, hence on the clinical phenotype, requiring diverse treatment options [11]. In fact, GoF has been detected in variants from either *SCN2A*-related DEE or BFNE phenotype; the plausible reason may be that the pathogenic variants detected in BFNE increase the neuronal excitability during early development but not in the mature brain. The variants detected in DEE may instead result in a greater degree of neuronal excitability that persists in the mature brain. In contrast, opposing effects of LoF on human voltage-gated sodium channel Nav1.2 function leading to a reduction in neuronal excitability in pyramidal neurons have been detected in the majority of missense variants associated with autism [12]. The neuropathological mechanisms defining why both GoF and LoF could lead to DEE phenotype remain unclear. However, the distinct phenotype difference between GoF and LoF should be investigated to guide precise treatments, because patients with LoF variants should avoid sodium-channel blockers (SCBs) [13].

Early and targeted genetic testing would be desirable to become a widespread routine procedure. To identify the different clinical pictures and specific genetic causes, discriminating between Lof and GoF variants, may be crucial to guide appropriate therapeutic and prognostic decisions [1,8,14].

## 2. Epileptogenesis in the Neonatal Brain

Epileptogenesis is a process through which a wide variety of causes (genetic, structural, metabolic, etc.,) may determine changes in the neuronal tissue, leading to the generation of unprovoked seizures or their progression after the epileptic condition has been established [5,15]. This process occurs during the early stages of life, hence, in newborns and infants [2]. These specific populations present some age-specific peculiarities, responsible for a higher seizure susceptibility in comparison to other phases of life, among which are different synaptic maturation and degree of myelination, different neuronal network properties and functions, as well as ion homeostasis and characteristic patterns of neurotransmitters expression and signalling [5]. In this regard, the developing brain presents a unique arrangement of GABA and glutamate receptors [5]. In particular, in the adult brain, GABA ionotropic receptors (GABA_A_R and GABA_C_R) usually determine an influx of Cl^−^ ions into the neuronal cell, leading to its hyperpolarisation; while, in newborns, the activation of these receptors causes opposite effects. This is due to a different distribution of the cotransporters of Cl^−^, with a prevalence of potassium-chloride cotransporters (KCCs) in the developing brain, as opposed to the dominance of sodium-potassium-chloride cotransporters (NKCCs) in the mature one. In fact, the former is responsible for reducing intracellular levels of Cl^−^, in contrast to the latter, which determines Cl^−^ increase into the cell. In other words, the resulting Cl^−^ gradient of the immature brain (Cl^−^ higher in the intracellular compartment than in the extracellular one) ensures that the activation of GABA receptors produces the efflux of Cl^−^ through the cell membrane, resulting in a neuronal depolarisation [16,17,18]. Pathogenic variants of GABA-R subunits have been identified in several early-onset epilepsies, such as infantile spasms or West syndrome [18,19,20].

As far as the glutamate neurotransmission is concerned, two main types of receptors are of interest in the immature brain and, particularly, in the epileptogenesis of newborns, namely the NMDARs and AMPARs. It has been reported that these receptors manifest a different configuration in terms of subunits in the developing brain. Specifically, NMDARs present an increased amount of NR2B, NR2A and NR3A subunits, whereas AMPARs have been demonstrated to be lacking the GluR2 one [21]. These conformational changes are responsible for a higher calcium influx, resulting in a lower threshold for seizures [21]. Therefore, it may be concluded that the immature brain is per se particularly prone to developing epileptic attacks, especially when disease-causing variants occur, further impacting on a vulnerable system [5,22].

## 3. Epileptic Phenotypes of Newborns

According to the ILAE classification, three main groups of clinical epileptic phenotypes are traditionally recognised in newborns: BFNE, EME and OS [2,9]. This classification appears too simplistic, and it reflects a far more conspicuous number of clinical entities, extremely heterogeneous, though potentially sharing the same genetic cause (pleiotropy) [14,23]. Most of the genetic epilepsies do not fall in fact within these groups, being rather included in the category of DEE [14].

**Figure 1:**
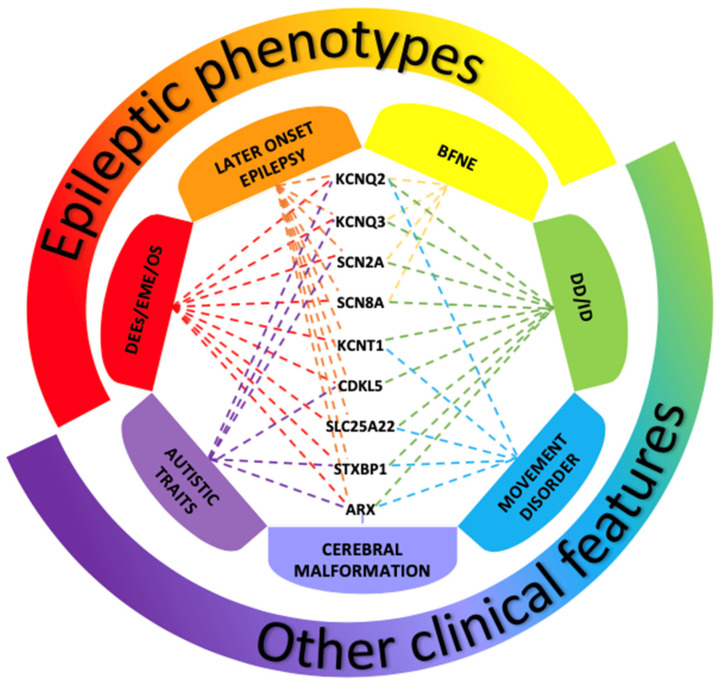
Disorders associated with variants of genes most frequently involved in neonatal-onset epilepsies. The picture shows the clinical phenotypes associated with variants of genes most fre-quently involved in neonatal-onset epilepsies. Benign familial neonatal epilepsy (BFNE) has been commonly linked to KCNQ2/3 genes, but also to SCN2A, and recently to a few cases of SCN8A variants. Pathogenic variants of all the reported genes have been related to severe forms of neo-natal-onset developmental and epileptic encephalopathies (DEEs), such as early myoclonic en-cephalopathy (EME) and Ohtahara syndrome (OS). Variants of these genes have also been associ-ated with other clinical presentations, including later-onset epilepsy, developmental de-lay/intellectual disability (DD/ID), movement disorders, autistic traits and cerebral malformations. The overlap of these clinical features makes it difficult to establish a precise genotype-phenotype correlation, delaying the assessment of the condition and the start of a proper treatment.

Owing to recent advances in research, many pathogenic variants have been associated with neonatal-onset seizures, and, nowadays, it is commonly accepted that plural genetic defects may lead to similar clinical features (genetic heterogeneity) [24], which may partly overlap (Figure 1) (2). Defining the genotype-phenotype correlation and the etiopathogenic mechanisms appears therefore fundamental to establishing a precise diagnosis and prognosis and, hopefully, achieving the conceiving of a prompt and targeted therapy [2].

### 3.1. Benign Familial Neonatal Epilepsy (BFNE)

BFNE is a genetic condition characterised by the onset of seizures during the first weeks of life in an otherwise healthy child with a normal neurological examination. Usually, this condition is due to pathogenic variants involving genes with autosomal dominant inheritance (coding for voltage-gated channel subunits). It is frequently expressed in more than one generation in the same family. A familiar history of neonatal seizures should indeed lead to suspicion of this disorder [25,26].

Notably, *KCNQ2* and, less frequently, *KCNQ3* genes are the most frequently considered causative of BFNE [25]. Specifically, the *KCNQ2* gene (located on chromosome 20q13.3) encodes the α-subunits of the potassium channel, which can interact with those produced from the *KCNQ3* gene (8q24) [5]. These subunits, forming functional potassium channels widely expressed within the brain, are responsible for producing the so-called neuronal M-current. This is a slow activating non-inactivating potassium current, crucial in the modulation of the resting membrane potential [27].

*KCNQ2/3*-related BFNE was first described in 1998 by Singh et al. [28]. This clinical entity usually presents with a wide variety of clinical seizures (mostly tonic or clonic with apnoeic episodes, and, less commonly, autonomic signs), occurring between the 2nd and 8th day of life, typically with a self-limiting course within weeks or months [14]. Neuroimaging exams and EEG do not show specific abnormalities, and the outcome in terms of neurodevelopment is usually good, though recurrence of epileptic episodes may still occur later in life [14,25].

LoF variants of *SCN2A* gene have also been brought into play in the genesis of BFNE. The *SCN2A* gene is located on chromosome 2q24.3 and encodes the major subunit of voltage-gated sodium channels involved in initiating and conducting excitatory action potentials [29]. Variants of *SCN2A* with GoF effects determine an increased neuronal excitability, with consequential implications in treatment choice [30].

Although *SCN2A* has been linked to other epileptic phenotypes, including developmental and epileptic encephalopathy (DEE) (see Section 3.2.2), BFNE was the first one to be described in relation to its pathogenic variants. *SCN2A*-related BFNE is a self-limiting autosomal-dominant disorder with neonatal onset of focal clonic, tonic and generalised tonic-clonic seizures, at times associated with motor manifestations, staring, and apnoea. Usually, interictal EEG results are normal or present focal or multifocal spikes on a normal background activity. The outcome is usually benign with a normal cognitive development and offset of seizures within early childhood [31].

It is noteworthy mentioning that, in light of the typical self-limiting course of BFNE, ILAE refers to this clinical entity as self-limited familial neonatal epilepsy [32].

### 3.2. Developmental and Epileptic Encephalopathies (DEEs)

Pathogenic variants of genes notably involved in BFNE may also determine more severe epileptic phenotypes presenting with developmental delay (DD), potentially evolving in intellectual disability (ID), and psychiatric and behavioural disorders. The epileptic activity may interfere with cognitive advancement, either in patients with previous physiological neurodevelopment or prior psychomotor delay [32]. However, it is fundamental to acknowledge that clinical phenotypes of DD/ID not associated with epileptic seizures or EEG abnormalities have also been related to variants of the same genes, suggesting a primary genetic implication in the origin of these disorders. Pathogenic variants of the same genes may determine different phenotypes of encephalopathies, which may include or not epileptic manifestations. For this reason, the ILAE proposed the term ‘developmental and epileptic encephalopathy’ (DEE) to classify those conditions in which both the epileptic and the developmental components contribute to the clinical phenotype [32]. Within the framework of the DEEs, two conditions are of interest in the neonatal period, namely the early myoclonic encephalopathy (EME) and the Ohtahara syndrome (OS), characterised by specific electroclinical patterns. Most of the neonatal-onset DEEs do not reflect the criteria of these electroclinical syndromes and are, therefore, referred to as unclassified DEEs [14].

#### 3.2.1. Early Myoclonic Encephalopathy (EME)/Ohtahara Syndrome (OS)

A clear distinction between EME and OS is difficult to make because these two electroclinical conditions frequently present a big overlap in terms of both clinical and electric features [33].

EME is a severe epileptic condition affecting newborns in the first month of life. It is often considered as the result of metabolic causes, although cerebral malformations and non-metabolic genetic causes have been reported as well [2,26]. Typical ictal manifestations include focal motor seizures, tonic spasms, focal or fragmented myoclonus and massive myoclonias. Usually, the EEG shows a background activity consistent with a burst-suppression (BS) pattern, with bursts of spikes and sharp waves lasting for 1–5 s, alternating with flat periods of 3–10 s, mostly during sleep, potentially resulting in atypical hypsarrhythmia later in life [22,26]. Generally, EME is a serious and drug-resistant encephalopathy, leading to death within the first year of life in 50% of cases, and with an invariable severe neurodevelopmental outcome in those that survive [26].

OS is considered a genetic disease, although it has also been related to cortical malformations, such as porencephaly, lissencephaly, and Aicardi syndrome [26]. Onset is during the first three months of life, presenting with frequent tonic spasms (100–300 *per* day), often grouped in clusters, and, rarely, focal seizures. The EEG shows a typical continuous BS pattern, both in wake and sleep, at times asymmetric [22,26]. The prognosis is severe, though usually better than EME, and may evolve into infantile spasms [26].

#### 3.2.2. Genes and Pathogenic Variants Mostly Involved in DEEs

Regardless of the potential plurality of causes (i.e., metabolic, genetic and/or structural defects), it is commonly accepted that a monogenic disease-causing variant might be the actual primary causative factor of both EME and OS, but also of other neonatal-onset unclassified DEEs [2,8,14]. To date, many genes have been brought into play, such as *KCNQ2*, *KCNQ3*, *ARX*, *STXBP1*, *SLC25A22*, *CDKL5*, *KCNT1*, *SCN2A* and *SCN8A* [2,14,25,34].

Particularly, pathogenic *KCNQ2* and *KCNQ3* variants permit to emphasise how different variants of the same gene can result in different, and even contrasting, epileptic phenotypes, ranging from the BFNE to severe DEE. *KCNQ2/3*-related DEE is mostly due to de novo missense variants causing a dominant-negative effect or a loss of function of the M-current. However, parental mosaicism or rare variants leading to a gain of function of the M-current have also been reported [25].

The *ARX* gene is located on chromosome Xp21.3 and encodes a transcription factor involved in regulating neuronal migration and differentiation during brain development. Besides OS, it has also been related to the X-linked lissencephaly with abnormal genitalia (often involving neonatal seizures, or even prenatal ones) and Partington syndrome (a neonatal-onset disease leading to epilepsy, focal dystonia of the hands, intellectual disability and autistic traits) [14,35].

*STXBP1* gene (chromosome 9q34.11) encodes the MUNC18-1 protein, involved in the regulation of synaptic vesicle exocytosis [36].

*STXBP1*-related DEE is usually consistent with an OS phenotype, starting with tonic seizures, particularly drug-resistant, which may end up into infantile spasms within the first months of life [37].

Among the other possible clinical presentations, *STXBP1* pathogenic variants may generate focal seizures with neonatal-onset, non-syndromic epilepsies, Rett syndrome and intellectual disability without epilepsy [38]. Overall, although it has been linked to a wide variety of developmental and epileptic phenotypes, disease-causing variants of *STXBP1* almost invariably present with developmental delay and intellectual disability [36].

The *SLC25A22* gene (chromosome 11p15.5), namely the Solute Carrier Family 25, Member 22, also known as *GC1* gene, belongs to the *SLC25* gene family, involved in the transmembrane mitochondrial transport of various metabolites [39]. Nowadays, pathogenic variants of *SLC25A22* have been described as causative of plural forms of neonatal onset epileptic encephalopathies, such as EME and OS, but also malignant migrating partial seizures of infancy (MMPSI), at times manifesting peculiar clinical aspects, such as dyskinetic movements and oculogyric crisis [40].

The *CDKL5* gene is located on chromosome Xp22 and produces a large serine-threonine kinase essential for normal brain development and function [41]. *CDKL5*-related encephalopathy presents with an onset of epileptic spasms during the first three months of life and usually evolves in severe DEE and West syndrome [42,43]. CDKL-5 pathogenic variants may be responsible of a dramatic neurodevelopmental impairment, and present with autistic traits, ID and epilepsy [44].

*KCNT1* gene (9q34.3) encodes a sodium-activated potassium channel called Slack (sequence like calcium-activated potassium channel), relating to different forms of encephalopathy with onset within 6 months of age. The most frequently reported is MMPSI, with focal asynchronous intractable seizures and severe psychomotor delay. It also relates to autosomal dominant nocturnal frontal lobe epilepsy and other epileptic syndromes such as OS, EME, West and unclassified DEEs [45,46]. KCNT1 variants may also determine rare and complex phenotypes including, among the other conditions, severe dystonia, leukoencephalopathy and cerebellar ataxia and almost invariably, ID [47].

As previously mentioned, *SCN2A* disease-causing variants may be responsible of benign (self-limiting) disorders, but in most of the cases (60–70%), they may result in neonatal-infantile DEE with drug-resistant seizures and poor neurodevelopmental outcomes [31]. These cases are typically considered as a result of de novo missense heterozygous variants with a gain of function effects [30]. Particularly, two epileptic syndromes have been strongly associated with *SCN2A* variants, namely OS and MMPSI, though most of the cases manifest diverse unclassifiable epileptic phenotypes. Generally, at the onset of the disorder, seizures (which may be focal, tonic and tonic-clonic) are frequent and at times grouped in clusters. The EEG shows a slow and disorganised background activity with multifocal spikes. BS is rare and mostly present at the onset. Besides DEE, cases of intellectual disability, autistic traits and late-onset seizures have been reported too [31].

*SCN8A* is a gene located on chromosome 12q13.13, encoding for a voltage-gated sodium channel involved in the regulation of neuronal excitability. *SCN8A* pathogenic variants have been usually associated with a severe epileptic encephalopathy at very early onset, potentially leading to poor outcomes. Seizures may present with a different semeiology, including focal or generalised tonic-clonic ones, myoclonic absences or spasms, and, in most cases, these conditions are highly drug-resistant. EEG generally shows a diffuse background with slow activities and focal epileptiform discharges, mainly in the posterior regions [48]. However, disease-causing variants of *SCN8A* have been recently related to rare cases of BFNE, increasing the number of genes associated with a wide range of epilepsy phenotypes [49,50].

In addition, it is noteworthy mentioning that an increasing number of genes not traditionally considered as causative of neonatal-onset epilepsies or DEEs has been described. In this regard, a recent review shed light on the several genetic aetiologies of phenotypes with neonatal-onset epilepsies or DEEs and movement disorders, which often include genes typically considered as causative of later-onset disorders [10].

## 4. The Role of EEG in Neonatal Seizures

The frequency of seizures is highest in the neonatal period due to the greater susceptibility and excitability of the immature brain. Critical semeiology ranges from evident clinical signs, such as clonic movements or hypertonia, fine movements of the tongue or eyes, to more attenuated clinical manifestations. Sometimes seizures may not show an evident clinical correlation, hence making diagnosis more difficult. Numerous studies demonstrated that most neonatal seizures are clinically silent, and electrographic seizures are even more frequent in preterm infants [51,52]. In addition, even in those conditions initially presenting clinical manifestations, at times, the EEG and clinical signs can uncouple after treatment with first-line antiepileptic drugs (AEDs) [53,54]. Regarding this, the EEG appears to be the only tool to highlight any residual epileptic seizures and monitor the treatment’s effectiveness. Furthermore, the range of non-epileptic motor phenomena observed in the neonatal period is extensive, so the EEG is fundamental in avoiding misdiagnosis of seizures in newborns and also unnecessary pharmacotherapy [22]. EEG is also necessary to consider the differential diagnosis with movement disorders, often associated with some genetic epilepsies, such as those caused by *STXBP1*, *KCNQ2*, *SLC25A22* and *ARX* variants [2].

Many neonatal units widely exploit the amplitude EEG (aEEG), since it is more frequently available and easily interpretable even by non-experts, allowing to distinguish seizures from noisy signals caused by excessive biologic or environmental interference [22]. It should also be noted that the aEEG pattern of KCNQ2-related seizures is considered pathognomonic, since it fairly reflects the pattern seen on the full EEG, making the aEEG a useful tool to recognise these not so rare forms of genetic epilepsies [55].

Finally, the EEG is essential for detecting clinical and subclinical seizures, monitoring response to AED treatment, making a differential diagnosis and guiding the aetiology of the seizures. It turns out to be a unique biomarker of brain electrical activity, helping physicians in choosing the proper therapeutic approach and assessing the outcome [22].

## 5. Treatment

Early identification and treatment are crucial in managing neonatal seizures to avoid detrimental consequences on neurodevelopment, especially when associated with severe clinical phenotypes.

Several options are currently used to treat neonatal seizures, although one of the major issues regards the lack of effective antiseizure drugs. None of the most commonly used AEDs gives fully satisfying response rates, and some of them raise concerns because of their serious side effects [4].

Furthermore, the lack of a general consensus on the criteria to assess antiseizure medication efficacy in neonates, the paucity of randomised clinical trials and the very few drugs licensed to treat this population, make it all more complicated [4,56,57].

Conversely to epilepsy in adolescents and adults, epileptic manifestations in neonates and infants pose unique challenges, such as the variety of clinical onsets (including mere electric seizures) and aetiologies, which make the diagnostic process particularly difficult. The underlying aetiology of seizures should be the first aspect to consider when treating neonates. For instance, some early onset epilepsies (i.e., metabolic or symptomatic ones) may require precise types of intervention, such as ketogenic diet or surgery [56]. Similarly, pathogenic variants of early onset epileptic phenotypes may influence treatment choice in primary genetic conditions [4].

Early genetic testing must be evaluated when a genetic cause is suspected since precision genetic diagnosis enables precision therapy approaches [14]. The increasing number of variants involved in neonatal epilepsies, together with the rising interest and research on the topic, is slowly posing the basis for future individually tailored intervention strategies. In this regard, some genes may be responsible for typical phenotypes so that the semeiology per se may be suggestive of the aetiology and, when genetic, even of specific disease-causing variants [4,5,6,7,8,9,10,11,12,13,14,15,16,17,18,19,20,21,22,23,24,25,26,27,28,29,30,31,32,33,34,35,36,37,38,39,40,41,42,43,44,45,46,47,48,49,50,51,52,53,54,55].

Regarding *KCNQ2*-related epilepsies, clinical signs and symptoms suggestive of *KCNQ2* pathogenic variants (either determining benign or severe phenotypes) may help since these conditions may strongly benefit from the administration of sodium channel blockers (SCBs). Though Phenobarbital (PB) is still used as first-line therapy in newborns, Carbamazepine has been proved to be more efficacious, and it is strongly recommended when LoF-*KCNQ2* implication is suspected [58]. In a recent study and consistent with previous reports, Cornet et al. proved that PB is generally ineffective in neonatal genetic epilepsies [55]. In addition, increased neuronal apoptosis/toxicity and impairment in normal neonatal synaptic maturation have been reported in pre-clinical and clinical trials [58,59], and PB and Phenytoin use in neonates has also been proved to increase the electroclinical uncoupling phenomenon and to be associated with higher cognitive and behavioural side effects than newer antiepileptic drugs [54,56].

It should also be noted that in 2011 retigabine, a specific potassium channel opener, was approved by Food and Drug Administration as adjunctive therapy in adults with partial-onset seizures. Retigabine proved to be beneficial in specific pathogenic LoF variants of KCNQ2, but its effect remains uncertain in GoF variants [60,61]. This drug has been later withdrawn due to some side effects, but its first approval remains anecdotal to realise the importance of personalised therapies and underline the potentialities that future research may bring to light [62].

With respect to other precision therapies, SCBs may also be considered the optimal therapeutic choice when GoF *SCN2A*/*SCN8A* neonatal epilepsies are suspected, while controversial data have been reported in relation the use of quinidine in GoF *KCNT1* variants [14,63].

Among the first-line treatment, Levetiracetam (LEV) has been increasingly used in neonatal seizures throughout the past decade as an alternative first-line treatment, given its favourable pharmacokinetic profile and the few side effects, which make it particularly beneficial in a wide range of ages and conditions [56]. Recently, its use has been proved beneficial in patients carrying pathogenic variants of *STXBP1*, leading in some cases to full epilepsy control and EEG normalisation [64,65]. In consideration of all these useful aspects, Brivaracetam (BRV), deemed the second generation of LEV, has been considered a valid option to treat neonatal seizures [66]. In fact, BRV presents a higher affinity and selectivity for SV2A glycoprotein and less significant adverse events than LEV (among which irritability, dizziness, anxiety and the occurrence of status gelasticus or gelastic seizures) [67,68,69]. Moreover, observational studies reported an effective response also in patients with encephalopathic epilepsies of the early infancy (i.e., Lennox Gastaut Syndrome, Dravet Syndrome, juvenile myoclonic and myoclonic-atonic epilepsy), validating the use of BRV in epilepsy regardless of the aetiology [70,71]. In light of this, a clinical study is currently ongoing to evaluate the pharmacokinetics of brivaracetam (BRV) in neonates with seizures not adequately controlled with previous AEDs (ClinicalTrials.gov Identifier: NCT03325439; https://clinicaltrials.gov/ct2/show/NCT03325439, accessed date: 8 August 2021).

To date, other drugs are available and variably used, depending on the individual patient characteristics and/or on the centre experience [57]. In particular, in case of drug-resistant seizures or recurrent seizures evolving into status epilepticus, corticosteroid therapies have been proved to be a valid choice. In particular, adrenocorticotrophic hormone (ACTH) showed some temporary effect in patients affected by OS [20]. Whereas, more recently, hydrocortisone (5 mg/kg/day) showed efficacy in three patients with neonatal status epilepticus that failed conventional first- and second-line antiepileptic therapies [72].

## 6. Discussion

Seizures are frequent and dramatic neurological conditions in newborns, potentially due to acute events or structural, metabolic or genetic causes [1,8]. Regarding the latter, the interest in the topic is increasingly growing, and more research and studies are targeting new genetic aetiologies of neonatal-onset seizures [14]. Several genes have been demonstrated to be involved in the onset of neonatal seizures and epilepsy in general [14]. Most of them are widely and variously implicated in key phases of brain development and in the regulation of neuronal excitability, which is per se particularly high in the early stages of life. Therefore, pathogenic variants of these genes may further perturb the susceptible immature brain, making it more prone to developing epileptic attacks, which, in their turn, may be responsible for poor neurodevelopmental outcomes. Usually, seizures are very heterogenous in this period of life, though the prevalent types in the neonatal age are tonic, myoclonic or spasms [10]. Clinical semiology is essential since it may reflect the aetiology. In an observation double cohort study, Cornet et al., concordant to previous studies, demonstrated that focal and electric seizures are more often of acute provoked origin, tonic seizures are mostly related to genetic causes and myoclonic ones represent the most frequent type of seizures in patients with inborn errors of metabolism. These findings are significant since they may potentially guide treatment, not only while waiting for genetic testing results but also whenever the access to genetic testing is limited [55].

Today, a clear genotype-phenotype correlation is lacking. In fact, the phenotype spectrum associated with variants of a single gene may differ greatly, ranging from self-limiting forms of epilepsies to dramatic phenotypes of DEE. The difficulty in providing a clear phenotype-genotype correlation often makes the diagnostic process more complex, potentially compromising the choice of diagnostic tests, delaying the assessment of the condition and starting a proper treatment [2].

Moreover, the therapeutic process is further complicated because no specific protocols are available to manage specific epileptic conditions affecting newborns. Several drugs are available and commonly used, and a general agreement exists on first- and second-line therapies. However, the drug resistance of some severe conditions poses great challenges to physicians, requiring further interventions and plural pharmacological approaches, not always successful [4].

A recent paper by Bayat et al. [14] describes the electroclinical epilepsy syndromes occurring in the first year of life and the usefulness of genetic testing for adequate treatment. The increasing evidence available in genetics is indeed posing the basis for the development of new targeted therapies that may improve the prognosis of more severe phenotypes. For instance, the positive response of *KCNQ2*-epilepsies (regardless of the severity of the clinical phenotype) to SCBs may reflect the importance of choosing the treatment based on genetic diagnosis [14,25].

The development of new diagnostic and therapeutic algorithms could allow to reach a prompt diagnosis, detect the existence of specific genotype-phenotype correlation and lead to new targeted therapies.

## Data Availability

Not applicable.
